# Foreign Correspondence

**Published:** 1889-02

**Authors:** 


### Foreign Correspondence.

To the Editor:

Your letter of Oct. 28th received yesterday, and I need hardly say that if I can be of any service as Brazillian correspondent of the International Dental Journal, I will be happy to render any assistance in my power to forward the interests of what is, I trust, already an assured success. With best wishes believe me,

Yours very sincerely,

Sáo Paulo, Brazil, Dec., 1888. C. R. Fletcher.

---

To the Editor:

I beg to acknowledge receipt of your note of the 2d inst., and desire to state that, while expressing some conscious pride in your suggestion, I accept with pleasure the position of foreign correspondent, and will use my best endeavor to further the interests of the journal as it lies in my power so to do.

Jan. 7, 1889. J. Cowan Woodburn,
197 Bath street,
Glasgow, Scotland.

---

To the Editor:

I beg to acknowledge with thanks the receipt of your letter, and will be glad to accept the post of foreign correspondent to your journal. I think that there is a good field for an international high class journal, which shall be free from the petty personalities that are apt to disfigure American journals.

January 11, 1889. W. St. G. Elliott,
39 Upper Brook street, London, S. W.

